# Choroidal Venous Pressure Is Passively Controlled by Vortex Vein Collapse: A Theoretical Model

**DOI:** 10.1167/iovs.67.12.47

**Published:** 2026-10-22

**Authors:** Federica Vanone, Peter S. Stewart, Alexander J. E. Foss, Tamsin A. Spelman, Jennifer H. Tweedy, Rodolfo Repetto

**Affiliations:** 1 Gran Sasso Science Institute, L’Aquila, Italy; 2 School of Mathematics & Statistics, University of Glasgow, Glasgow, United Kingdom; 3 Department of Ophthalmology, Nottingham University Hospitals NHS Trust, Nottingham, United Kingdom; 4 Sainsbury Laboratory, University of Cambridge, Cambridge, United Kingdom; 5 Department of Mathematical Sciences, University of Bath, Claverton Down, Bath, United Kingdom; 6 Department of Civil, Chemical and Environmental Engineering, University of Genoa, Genoa, Italy

**Keywords:** mathematical modelling, starling resistor, vortex vein collapse, pressure regulation, choroidal circulation

## Abstract

**Purpose:**

Animal experiments have shown that choroidal venous pressure is tightly linked to the IOP. It has been hypothesied that the choroidal pressure is passively controlled by partial collapse of the vortex veins. This work aimed to test the feasibility of this hypothesis using mechanical modeling.

**Methods:**

We developed a theoretical model of venous outflow from the choroid as a Starling resistor. Each vortex vein was modeled as a collapsible tube formed from two segments arranged in series: one segment within the choroid, subject to an external pressure equal to the IOP, and another segment through the sclera subject to an external pressure, which is linearly decreasing from the IOP to the orbital tissue pressure. For a given choroidal blood flow, this model predicts the choroidal venous pressure and vortex vein cross-sectional area along its length. This model was validated against in vivo rabbit experiments, where the flow rate through the vortex vein was measured as the outlet venous pressure was adjusted.

**Results:**

In agreement with previous experiments, for constant choroidal blood flow, an increase in the IOP generates an almost equal increase in the blood pressure in the choroidal veins. Furthermore, the blood flow rate is insensitive to the outlet venous pressure provided it remains below the IOP. This model shows that partial collapse of the vortex veins explains both observations.

**Conclusions:**

This model provides a mechanical description of how choroidal venous pressure is passively controlled by vortex vein collapse. The model also shows that vortex vein collapse is expected within the sclera.

Recent recognition of the pachychoroid spectrum of disorders has brought renewed interest in the choroidal circulation and particularly the choroidal venous flow. The choroid is a densely vascularized layer of the eye, lying between the retina and the sclera, and has the highest blood flow per unit mass of tissue in the human body. The choroidal circulation originates from branches of the ophthalmic artery that enter the eye by piercing the sclera around the optic nerve. The choroidal arteries feed a very dense sheet of capillaries, known as the choriocapillaris. Venous drainage in the human choroid occurs typically through three to eight vortex veins,^1^^,^^2^ which exit the eye by passing through the sclera, and drain into the superior and inferior ophthalmic veins.

This circulation exhibits several unusual characteristics compared with most other vascular beds. The capillaries of the choriocapillaris are significantly larger than typical capillaries, with diameters ranging from 15 to 22 µm, whereas most systemic capillaries measure approximately 5 to 7 µm.^3^^–^^6^ In addition, the choroidal circulation is characterized by an exceptionally high blood flow. Oxygen extraction is unusually low, with only about 5% of the oxygen delivered by the choroidal blood supply extracted, as compared with a typical value of 30% for other systemic circulations.^7^^,^^8^ Moreover, the blood within this circulation also has a relatively high hematocrit. Furthermore, the vascular network includes venous–venous anastomoses, and these contribute to its distinctive structural and functional organization.^4^

The pachychoroid disorders spectrum, which includes central serous retinopathy, are characterized by a thickened choroid and enlarged vessels in Haller’s layer that can be up to 300 µm in diameter.^9^ These vessels are generally thought to be veins on basis of indocyanine green angiography and optical coherence tomography angiography, which show dilated choroidal veins converging into an ampulla adjacent to the sclera.^10^^,^^11^ This has lead to the proposal that various clinical features seen in central serous retinopathy can be explained by choroidal venous congestion and these findings have been reproduced in an animal model where two of the vortex veins of a monkey’s eye were ligated.^12^

Under physiological conditions, the choroidal venous pressure is approximately 2 mm Hg higher than the IOP. Animal experiments have shown that this pressure offset remains almost constant when the IOP is artificially raised, even for values of up to 100 mm Hg, demonstrating a tight link between the choroidal venous pressure and the IOP.^13^^,^^14^

Furthermore, Bill^15^ performed experiments on rabbits in which he cannulated a vortex vein, imposed a prescribed downstream pressure, and measured the blood flow rate through that vein. As the downstream pressure was increased starting from the atmospheric pressure, the flow rate remained approximately constant until the pressure reached a value close to the IOP. As the pressure was increased further, the flow rate progressively decreased until, for sufficiently high values of the imposed pressure, it ceased entirely. These observations suggest the existence of a pressure- and flow-regulation mechanism controlling the choroidal blood outflow.

A possible explanation is the autoregulation of the choroidal blood vessels. Choroidal arterial perfusion exhibits autoregulation in response to changes in arterial blood pressure^16^^–^^18^; however, no comparable autoregulation has been observed in the choroidal veins, and therefore this mechanism cannot explain the above observations. Bill^15^ proposed a purely passive control mechanism, arguing that his experimental findings could be explained by assuming that the scleral segment of the vortex veins is normally in a state of partial collapse. The possible role of the collapse of the vortex veins in regulating choroidal venous pressure was also discussed by Spaide et al.,^19^ who compared the vortex vein system with a Starling resistor.

A Starling resistor is a simple experimental setup involving flow through a segment of flexible tubing mounted between two rigid pipes, which can be manipulated manually to vary the flow resistance.^20^ Over the years, this setup has become a canonical experiment in its own right, useful for studying phenomena such as flow limitation and self-excited oscillations.^21^^–^^24^ These experimental setups have also been studied using theoretical and computational models, making some progress toward a mechanistic understanding.^25^^–^^28^ However, compared with other Starling resistor systems, the vortex vein has the peculiar feature that it is subjected to an external pressure that varies spatially as it leaves the eye through the sclera. Indeed, the external pressure acting on the vessel is constant and equal to the IOP inside the choroid. Across the scleral segment, we assume it to decrease due to water efflux across the scleral tissue, until it becomes equal to the orbital tissue pressure outside the eye (see the External Pressure Acting on the Vortex Vein section for details).

In this work, we propose a simple model of steady flow through the vortex vein as a Starling resistor, aimed at identifying feasible mechanical mechanisms that justify the experimental observations described. In particular, we aimed to clarify the role that vein collapse has on the tight link between the IOP and choroidal venous pressure. We also consider the possibility of venous collapse across the intrascleral segment.

## Methods

### General Description of the Mathematical Model

We consider the flow of blood along a single vortex vein that originates in the choroid and exits the eye through the sclera. We consider an axis along the center of this vortex vein spanned by a coordinate *z*. We assume that this vortex vein is formed from two segments in series, an upstream segment of length *L*_*c*_ within the choroid itself, and a downstream segment of length *L*_*s*_ through the sclera; we set the origin of the coordinate *z* at the boundary between choroid and sclera (see Fig. 1). Thus, our domain is the region −*L*_*c*_ ⩽ *z* ⩽ *L*_*s*_ and the point *z* = *L*_*s*_ denotes the exit of the vortex vein from the eye into the orbit.

**Figure 1.**
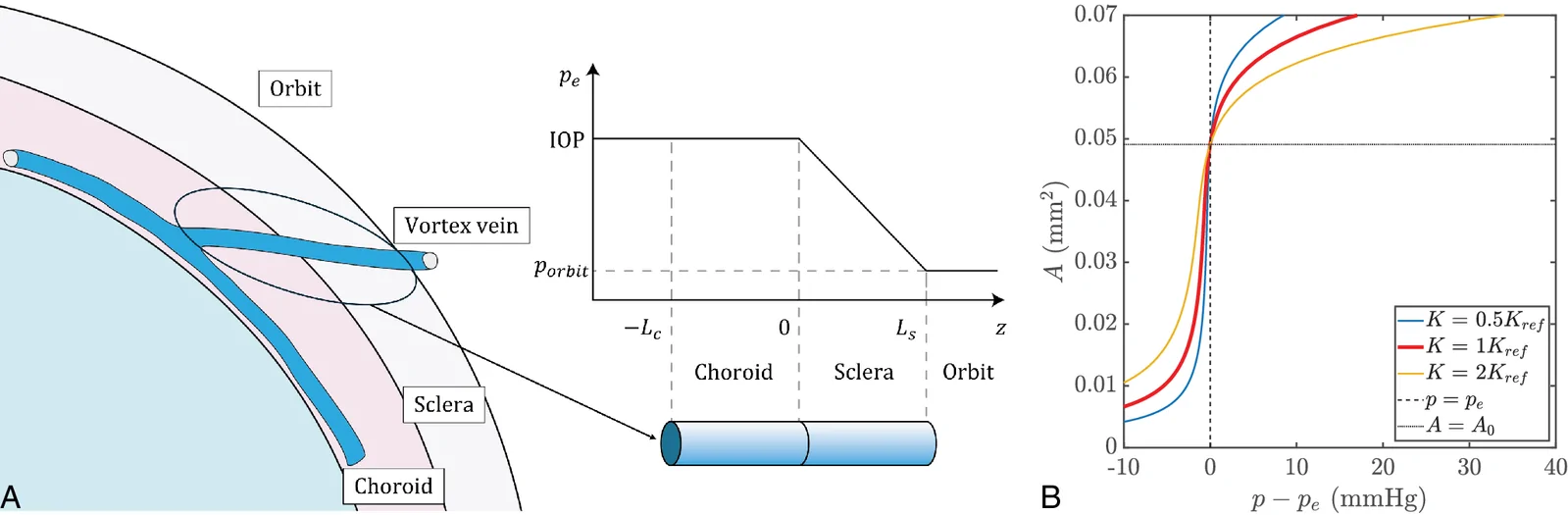
(**A**) On the left, sketch of the essential elements of the system we are considering: the vortex vein collects blood from the choroidal circulation and drains it out of the eye (orbit). The two parts of the vortex vein we consider in the model are the choroidal and scleral segments. On the right, the domain of the model: the axis of the tube representing the vortex vein starts in −*L*_*c*_ and ends in *L*_*s*_. The external pressure *p*_*e*_(*z*) acting on the tube is IOP in the choroid, and it decreases linearly to *p*_*orbit*_ in the sclera. (**B**) Plot of the tube law (3) for three different values of stiffness *K*. The other parameters are set as in the Table for humans.

### External Pressure Acting on the Vortex Vein

The vortex vein exits the eye passing through the sclera. Within the choroid, the external pressure *p*_*e*_(*z*) acting on the vessel is the IOP, whereas in the orbit it is the orbital tissue pressure *p*_*orbit*_; both the IOP and the *p*_*orbit*_ are assumed to be uniform and constant. If we consider the sclera as a low-permeability porous medium,^29^ we can assume that there is a very slow interstitial fluid flow from the choroid to the orbit through the sclera. This flow is influenced by solutes present in the fluid, notably albumin,^30^ but for simplicity we neglect this here. Since the scleral thickness is much smaller than the radius of the eye, we may assume that the fluid flow in the sclera is uniform in the radial direction and thus constant across the thickness of the sclera. This means that the pressure within the sclera must vary linearly from the IOP at the boundary with the choroid to the orbital pressure outside the eye:

$$\begin{array}{c} p_{e}\left(z\right)=p_{orbit}-\left(\text{IOP}-p_{orbit}\right)\frac{\left(z-L_{s}\right)}{L_{s}},\;\left(0\leq z\leq L_{s}\right), \end{array}$$

as plotted in Figure 1A.

### Fluid Flow in the Vortex Vein

We modeled the vortex vein as a long collapsible tube with a cross-sectional area, *A*, that can vary along the *z*-axis, with a uniform baseline value *A*_0_. We assume the vessel cross-sectional area is circular in its equilibrium configuration, with a baseline diameter of *d*_0_, corresponding with the baseline area $A_{0}=\frac{1}{4}\pi d_{0}^{2}$. We also assume that the vein cross-section remains circular as the vessel expands (*A* ⩾ *A*_0_), while it becomes elliptical in the collapsed case (*A* < *A*_0_).^31^

The flow in the vortex vein is characterized by relatively small values of the Reynolds number (the ratio of inertial to viscous effects in the flow); based on the values reported in the Table for human, one obtains Re = ρ*U*_0_*d*_0_/µ ≈ 4, where *U*_0_ is the cross-sectionally averaged blood velocity and ρ and µ are blood density and viscosity, respectively.

**Table.** Parameter Values and References

Parameter	Description	Value	Reference
*d*_0_	Baseline diameter of the vortex vein:		
	– human	250 µm	Cai et al.^39^
	– rabbit	200 µm	Assumed
*U*_0_	Baseline average velocity	7 cm/s	Abdallah et al.^40^
*q*	Flow rate in a single vortex vein	200 µL/min	Estimated as $q=\frac{\pi }{4}d_{0}^{2}U_{0}$
*L*_*s*_	Length of vortex vein in the sclera:		
	– human	4000 µm	Imanaga et al.^41^
	– rabbit	2500 µm	Estimated (Parameter values Section)
µ	Blood viscosity	0.0042 Pa · s	
ρ	Blood density	1060 kg/m^3^	
*p*_*orbit*_	Orbit tissue pressure:		
	– human	3.5 mm Hg	Enz and Tschopp^42^
	– rabbit	3.5 mm Hg	Forrest et al.^43^
*n*, *m*	Exponents of the tube law	1.5, 10	Toro et al.^34^
*K*_ref_	Vein stiffness parameter, baseline value	66 Pa	Estimated (Parameter values Section)

When different parameters are used for rabbit and human, this is specified. Details are included in the Supplementary Material, Parameter values.

The IOP varies periodically with the cardiac cycle, and this might influence blood flow in the vortex vein. The typical time scale of the heartbeat is ≈1 second; in the Supplementary Material, we show that the time required for a flow in a pipe with the diameter of the vortex vein to adapt to the instantaneous value of the pressure gradient is three orders of magnitude smaller. Thus, we can model the flow in the vortex vein as approximately steady, instantaneously adapted to the pressure gradient. We assume that, in the range of the IOP considered, the blood flow is constant. Indeed, considering possible changes of perfusion flow rate in response to changes in the IOP would require accounting for choroidal arteries, choriocapillaries, and autoregulatory mechanisms, which is beyond the scope of this work.

In general, the flow of blood along the vessel can be assumed to follow the Navier–Stokes equations.^32^ However, assuming the variations in cross-sectional area occur over a length much longer than the vessel diameter, the Navier–Stokes equations can be reduced to a single equation for the pressure *p*(*z*) along the vessel (see the derivation in the Supplementary Material):

(1) $$\begin{array}{c} \frac{dp}{dz}=-Rq,\;\left(-L_{c}\leq z\leq L_{s}\right),\; \end{array}$$

where R is a friction term that depends on *A*, *A*_0_, and μ. In our model, R is computed based on the assumption that the vein maintains a circular cross-section when inflated (*p* > *p*_*e*_), while its shape becomes elliptical during collapse^32^^,^^33^ (*p* < *p*_*e*_):

(2) $$\begin{array}{c} R=\left\{\begin{array}{c} \frac{8\mu \pi }{A^{2}},\;p\geq p_{e}\\ \frac{8\mu \pi }{A^{2}}{\left(\frac{A_{0}}{A}\right)}^{1/2},\;p\leq p_{e}. \end{array}\right.\; \end{array}$$

See Supplementary Material, Section 1.2. We solve this equation by imposing the venous blood pressure *p*_*o*_ at the outlet into the orbital region, so that *p*(*z* = *L*_*s*_) = *p*_*o*_. Under our assumptions, the flow rate *q* is imposed, and is constant along the tube. We note that if the diameter of the vessel is uniform, then Equation (1) reduces to the familiar Poiseuille law used to describe steady viscous flow along a rigid tube.^32^

Equation (1) is nonlinear since, for a collapsible tube, the vessel cross-sectional area *A* depends on the transmural pressure *p* − *p*_*e*_. The mechanics of the compliant vessel wall are captured through an elastic tube law, which relates the cross-sectional area *A* of the vessel to the transmural pressure across the vessel wall (*p* − *p*_*e*_). In this work, we follow Toro et al.^34^ and use the relationship for a compliant vessel:

(3) $$\begin{array}{c} p-p_{e}=K\left[{\left(\frac{A}{A_{0}}\right)}^{m}-{\left(\frac{A}{A_{0}}\right)}^{-n}\right],\;\left(-L_{c}\leq z\leq L_{s}\right),\; \end{array}$$

with the empirically determined values of the exponents for veins in general being *m* = 10, *n* = 3/2.^31^^,^^35^^,^^36^ This tube law is plotted in Figure 1B for different values of the parameter *K*, which describes the stiffness of the vein. For simplicity (and in absence of detailed measurements), we assume *K* to be uniform along the vessel. This tube law formulation dictates that the tube cross-section assumes its baseline area *A*_0_ when the transmural pressure is zero, that is, when *p* = *p*_*e*_.

We substitute *p* from Equation (3) in Equation (1) to get an ODE in the variable *A*, which we solve using the Matlab^37^ ODE solver ode15s, with the tolerance parameter RelTol set to 10^−7^. The solution is the cross-sectional area profile *A*(*z*) for −*L*_*c*_ ⩽ *z* ⩽ *L*_*s*_, from which we compute the blood pressure *p*(*z*) along the vessel length using the tube law (3).

In the Supplementary Material, Section 1, we report a complete derivation of the above equations and the estimates for the parameters in Table. In Section 2, we also formulate the fully nonlinear problem, including inertial effects, and compare the numerical results obtained with the two approaches, showing that the error on the pressure due to neglecting inertia is invariably <0.05%, even if the Reynolds number is increased up to Re ≈ 16, corresponding with a flow rate of 800 µL/min. The estimates made in this section are based on parameter values for humans, but similar results hold for rabbits.

### Modifications to the Model to Reproduce Bill’s^15^ Experiment

In this section, we describe how the model constructed above can be adapted to reproduce the experimental setup of Bill,^15^ where the author measured blood flow and pressure in a rabbit vortex vein. In these experiments, the choroidal venous pressure was measured in the segment of the vortex vein within the sclera (see Fig. 1 of Bill^15^). Also, the IOP was artificially regulated using a leveling manometer. One vortex vein was exposed and cannulated with a polyethylene tube outside the eye. Its downstream pressure was artificially increased stepwise by lifting the end of the tube and the resulting blood flow rate was measured within this vein.

To reproduce this experimental setup, we modified the model described in the previous sections as follows. We assume that choroidal blood flow is drained by four vortex veins,^38^ which are each identical to the model described above. These four veins are assumed to be connected with low-resistance shunts at their upstream ends within the choroid^15^; thus, in our model we arrange the four vessels in parallel and impose that they have the same upstream pressure. In three of these veins, the outlet pressure is kept constant and equal to its baseline value, while in the fourth the outlet pressure is progressively increased, reproducing the effect obtained by Bill in the cannulated vein. Furthermore, we impose that once the IOP is fixed, the total blood flow rate, given by the sum of the flow rates across all four vortex veins, is held constant. This hypothesis is based on the consideration that, when the flow through the cannulated vortex vein decreases due to the increased downstream pressure, the blood in the territory normally drained by the cannulated vein is shunted in the regions usually drained by the other veins.^15^

To obtain a solution, we solve Equations (1) and (3) in all four vessels, iterating the flow rate distribution in the vessels until the upstream pressure in all vessels is the same for the desired pressure outflow conditions (see Supplementary Material, Section 5).

For different values of the IOP, Bill^15^ measured different values of the baseline flow rate, that is, the flow rate in the cannulated vortex vein when the downstream pressure was set to the same value as that of the other uncannulated vortex veins. Thus, the total blood flow rate we impose in the model for each value of the IOP is four times the baseline flow rate measured by Bill in one vortex vein.^15^ We note that the parameter values used in this case are modified to reproduce the rabbit eye as in the Table.

## Results

### Pressure Distribution in the Vortex Vein and Relationship With the IOP

In this section, we show the predicted pressure distribution along the vortex vein and investigate its link to the IOP. All results in this section are obtained using parameter values for a human eye (Table), considering a choroidal segment of the vortex vein of the same length of its scleral segment *L*_*c*_ = *L*_*s*_.

Figure 2A shows pressure profiles *p*(*z*) along the vortex vein, for different values of blood flow rate *q*. All profiles are obtained by imposing the same value for the venous pressure at the outlet of the sclera, *p*_*o*_ = *p*_*orbit*_ + 2 mm Hg. The solid black line in the plot represents the external pressure *p*_*e*_(*z*) acting on the vessel, and the vertical dashed line marks the boundary between the choroid and the sclera. In Figure 2B we plot the corresponding profiles of the vessel cross-sectional area along its length. The black horizontal line corresponds with the vessel cross-sectional area in the fully open, relaxed state. The vessel is expanded if the area is above this value and partially collapsed if it is below.

**Figure 2.**
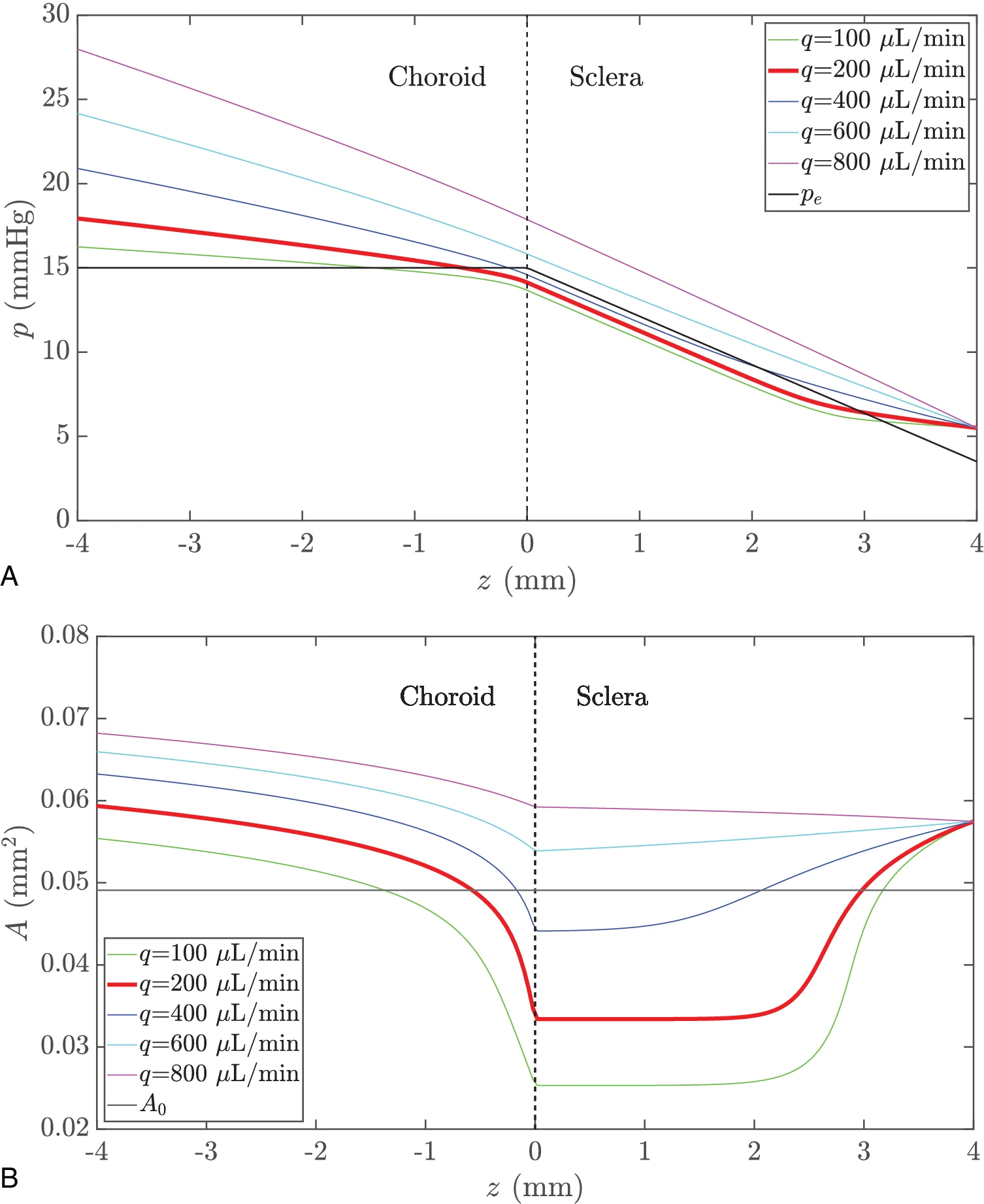
Pressure (**A**) and area (**B**) profiles for various values of the flow rate *q*. The other parameters are set as in the Table for humans. The dotted line at *z* = 0 is the boundary between choroid (*z* < 0) and sclera (*z* ⩾ 0).

For the largest values of *q* used in the plots (600 and 800 µL/min), the venous pressure is everywhere higher than the external pressure (*p* > *p*_*e*_) (Fig. 2A); therefore, the tube is expanded (Fig. 2B). Conversely, for smaller values of *q*, the venous pressure becomes lower than the external pressure somewhere within the sclera, causing a partial collapse of the vortex vein. Interestingly, in the partially collapsed cases, in the inner part of the sclera (away from the orbit), the fluid pressure *p*(*z*) closely follows the linear profile of the external pressure *p*_*e*_(*z*), having the same gradient, while the corresponding profile of the vessel cross-sectional area becomes uniform. Our estimate of the flow rate through a vortex vein is 200 µL/min (thick red curve in Fig. 2) and, thus, in normal conditions, the model predicts that the vortex vein partially collapses within the scleral segment, in agreement with Bill’s^15^ assumption.

In Figure 3, we fix the flow rate at the baseline value, *q* = 200 µL/min, and change the vessel wall stiffness *K* in the tube law (3) across a range which is sufficient to capture different qualitative behaviors. We show the corresponding pressure profiles (Fig. 3A) and vessel cross-sectional area profiles (Fig. 3B). We observe that the venous pressure *p*(*z*) follows the external pressure profile *p*_*e*_(*z*) in the scleral segment only if the vein is sufficiently compliant (low values of *K*; see *K* = *K*_ref_, *K* = 0.2*K*_ref_). For a stiffer vessel (*K* = 5*K*_ref_) with all other conditions held fixed, the profile is still partially collapsed within the sclera, but this is not sufficient for the vessel profile to become spatially uniform (Fig. 3B) or for the choroidal pressure to exhibit the same gradient as the external pressure (Fig. 3A).

**Figure 3.**
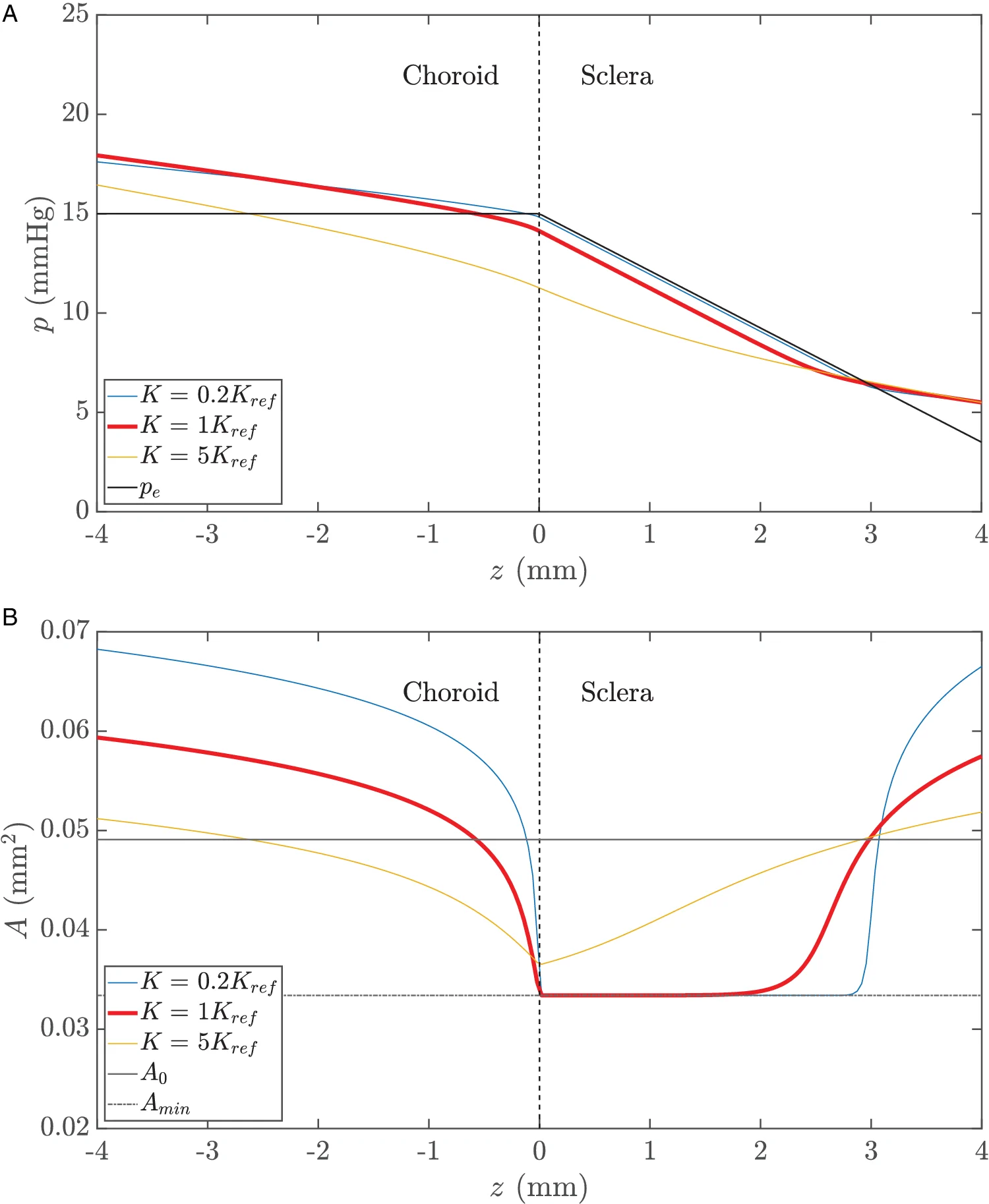
Pressure (**B**) and area (**A**) profiles for three values of stiffness *K*, where the baseline is *K* = *K*_ref_ = 66 Pa (thick red line). *K* = 0.2*K*_ref_ is softer, 5*K*_ref_ is stiffer than baseline. The dotted line at *z* = 0 is the boundary between choroid (*z* < 0) and sclera (*z* ⩾ 0). The other parameters are set as in Table for humans.

To gain further insight into the regime where the collapsed vessel adopts a uniform cross-sectional area, we reconsider Equation (1) and assume that the pressure gradient in the blood vessel exactly matches that in external pressure, so that d*p*/d*z* = −(IOP − *p*_*orbit*_)/*L*_*s*_, from which we compute the corresponding area as:

(4) $$\begin{array}{c} A_{min}={\left(\frac{8\pi \mu \sqrt{A_{0}}qL_{s}}{\text{IOP}-p_{orbit}}\right)}^{2/5}.\; \end{array}$$

This is the minimum value of the vessel area that satisfies the flow conditions. To understand why, we can inspect the pressure and area profiles shown in Figures 2 and 3. Starting from the downstream section and moving upstream, the transmural pressure decreases and so does the cross-sectional area; when the transmural pressure becomes negative, the vessel starts to collapse. As the area decreases in the upstream direction, the hydrodynamic friction increases according to Equation (2) and, therefore, the pressure profile becomes steeper, until it reaches exactly the slope of the external pressure, which corresponds to the minimal area *A*_*min*_ compatible with the flow. A further decrease in the area would make the pressure profile in the vessel steeper than the external pressure profile, and this would make the transmural pressure less negative, thus leading to an increase in the vessel area, which therefore cannot become smaller than *A*_*min*_.

Its value is plotted in Figure 3B as a dash-dotted black line. This formula demonstrates how, when the internal pressure profile is parallel to the external pressure in the scleral segment, the maximum possible degree of vessel collapse is set by the flow rate *q*, the length of the scleral segment *L*_*s*_ and the tissue pressure difference across the sclera (IOP − *p*_*orbit*_).

Animal experiments have shown that the IOP and choroidal venous pressure are tightly related: an increase in the IOP leads to an increase in the choroidal venous pressure of approximately equal magnitude.^13^^,^^14^ Figure 4 demonstrates how our model reproduces this effect, provided the vortex vein is sufficiently compliant. In Figure 4A, we plot the pressure at the interface between the choroid and the sclera *p*(*z* = 0) in terms of the imposed value of the IOP. In the figure, the flow rate is kept constant at its baseline value and the various curves correspond with different values of the vessel stiffness. When the vessel is sufficiently soft, this pressure is invariably very close to the IOP. However, this correlation is progressively lost as the vessel becomes increasingly rigid. The curve corresponding with the baseline value of stiffness is shown in thick red. By comparing Figures 3B and 4A, we note that the cases where *p*(*z* = 0) closely follows the IOP (*K* = *K*_ref_, *K* = 0.2*K*_ref_) in Figure 4A correspond with the cases for which the vein reaches its *A*_*min*_ within the sclera (Fig. 3B). Equation (4) thus gives a way to quantify the degree of collapse necessary to observe the tight correlation between the choroidal venous pressure and the IOP. For human baseline parameters and an IOP of 15 mm Hg, this is $\approx 100\frac{A_{min}}{A_{0}}\%\approx 68\%$. It is interesting to notice that *A*_*min*_ in Equation (4) is independent of *K*. In other words, the stiffness determines whether the vessel can collapse to its minimum cross-sectional area within the sclera, but it does not control the value of the minimum area itself.

**Figure 4.**
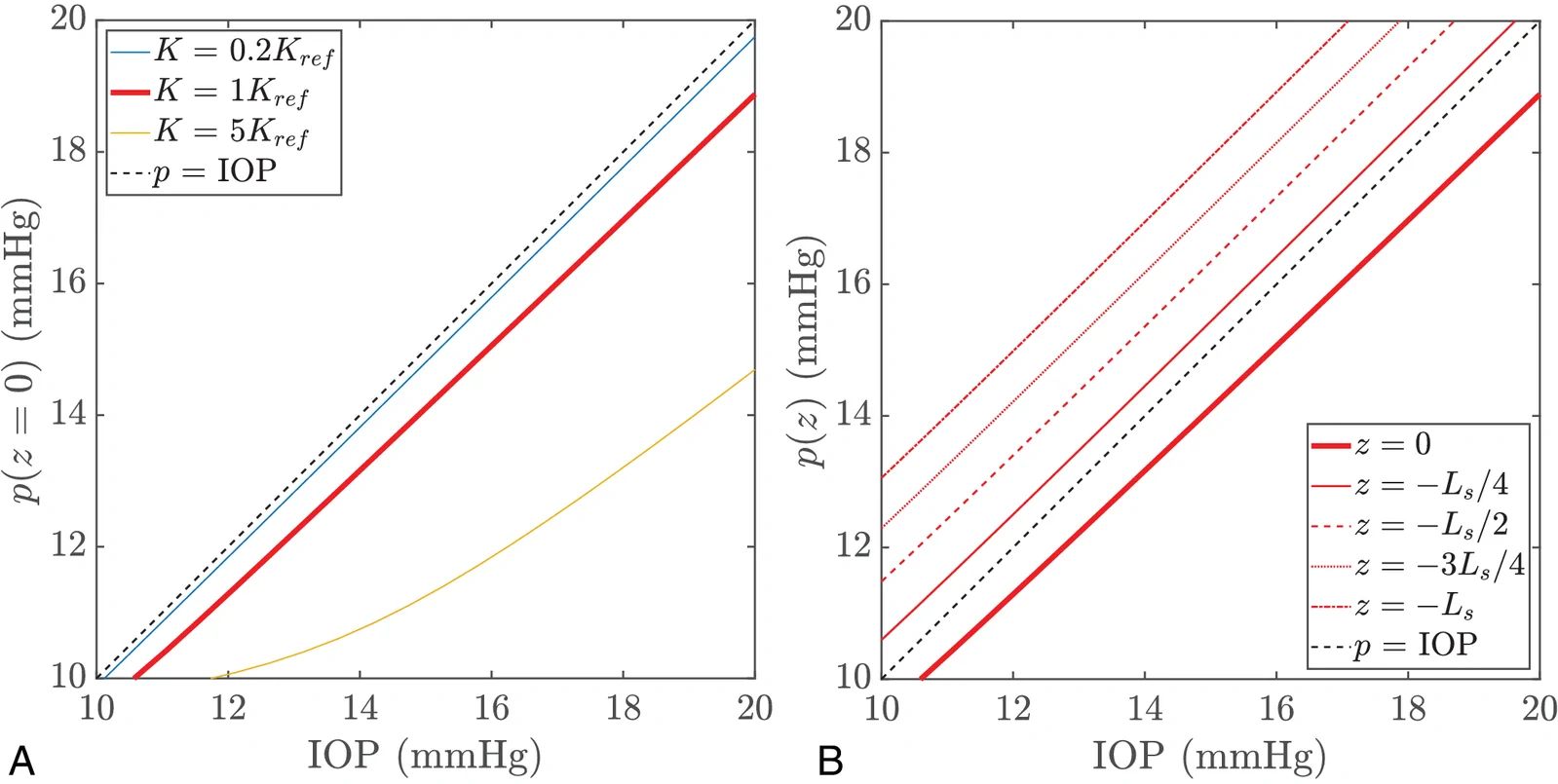
(**A**) Venous pressure at the inlet of the sclera (*z* = 0), plotted against the IOP for three values of the stiffness: the baseline value is *K*_ref_ = 66 Pa, *K* = 0.2*K*_ref_ is softer, and *K* = 5*K*_ref_ is stiffer than baseline. (**B**) Venous pressure at different locations from the inlet of the sclera (*z* = 0) to the inlet of the choroid (*z* = −*L*_*s*_), plotted against the IOP for the baseline value of stiffness *K*_ref_ = 66 Pa. In both panels, parameters other than *K* are set as in the Table for humans.

Furthermore, we can use Equation (4) to estimate the offset between the IOP and the vortex vein pressure (Δ*p*_*min*_) at the junction between the choroid and the sclera (*z* = 0), when the vessel is maximally constricted. Substituting *A* = *A*_*min*_ into (3) we obtain:

(5) $$\begin{array}{c} \Delta p_{min}=K\left[{\left(\frac{A_{min}}{A_{0}}\right)}^{m}-{\left(\frac{A_{min}}{A_{0}}\right)}^{-n}\right].\; \end{array}$$

To observe this estimated offset between the IOP and the blood pressure at the choroid–sclera boundary (*z* = 0), it is sufficient that the vessel is maximally collapsed (i.e., it reaches its minimum area *A*_*min*_) at that location.

Note that, in all cases considered in Figure 4A, the pressure at the boundary between the choroid and the sclera is below the IOP, and so the vessel is collapsed as it enters the sclera. However, Figure 4B shows that, for the baseline value of the vessel stiffness, the pressure within the vein soon becomes higher than the IOP as one moves upstream along the vessel, such that at a distance from the sclera of *L*_*s*_/4 = 1 mm, the pressure in the vein is already slightly higher than IOP. These results are in agreement with the findings of Bill^14^ and Mäepea,^13^ who reported a choroidal venous pressure about 2 mm Hg higher than the IOP, but did not specify the exact location where the venous pressure was measured and just indicated that the veins were within the choroid.

### Interpretation of Bill’s^15^ Experiment

Based on rabbit experiments, Bill^15^ argued that the vortex veins are in a partially collapsed state within the sclera. In this section, we use our model to reproduce their experimental results, as described in the Section Modifications to the Model to Reproduce Bill’s Experiment. In this section, variables with superscript *c* and *u* refer to the cannulated and undisturbed veins, respectively.

Figure 5A replots the experimental results of Bill^15^ (open circles), showing the blood flow rate in the vessel in terms of the outlet pressure $p_{o}^{c}$ in the cannulated vortex vein. The outlet pressure in the undisturbed veins is set to $p_{o}^{u}=p_{orbit}+2$ mm Hg. For each of the three values of IOP considered (three distinct colors), the flow rate in the cannulated vortex vein did not respond to the change in outlet venous pressure $p_{o}^{c}$ until the imposed pressure reached a value close to the IOP. For a further increase in the downstream pressure, the flow rate in the cannulated vein gradually decreased to zero. This behavior is very well reproduced by our model, as indicated with solid bold lines in the same color, which are obtained with our reference set of parameters for rabbit (Table). We emphasize that there is no fitting of any model parameter to reproduce Bill’s^15^ curves. We test the robustness of the results by varying the stiffness *K* in the range [*K*_*ref*_/2, 2*K*_*ref*_], obtaining the results in the shaded areas around the bold lines. The extremes of the range are the edges of the shaded regions. The decrease of the flux in response to the increase of $p_{o}^{c}$ is steeper for stiffer vessels. Similar tests on the robustness of the results with respect to the constant outlet pressure in the undisturbed veins $p_{o}^{u}$ and on the pressure in the orbit *p*_*orbit*_ are reported in the Supplementary Material, Section 6 and figure 3. Since, for each value of IOP, the total blood flow is kept constant in the model, when the flow starts decreasing in the cannulated vein, the model predicts that the flow must increase accordingly in the remaining veins. This is in line with Bill’s^15^ hypothesis that the blood flow normally drained by the cannulated vessel is shunted into the other vortex veins.

**Figure 5.**
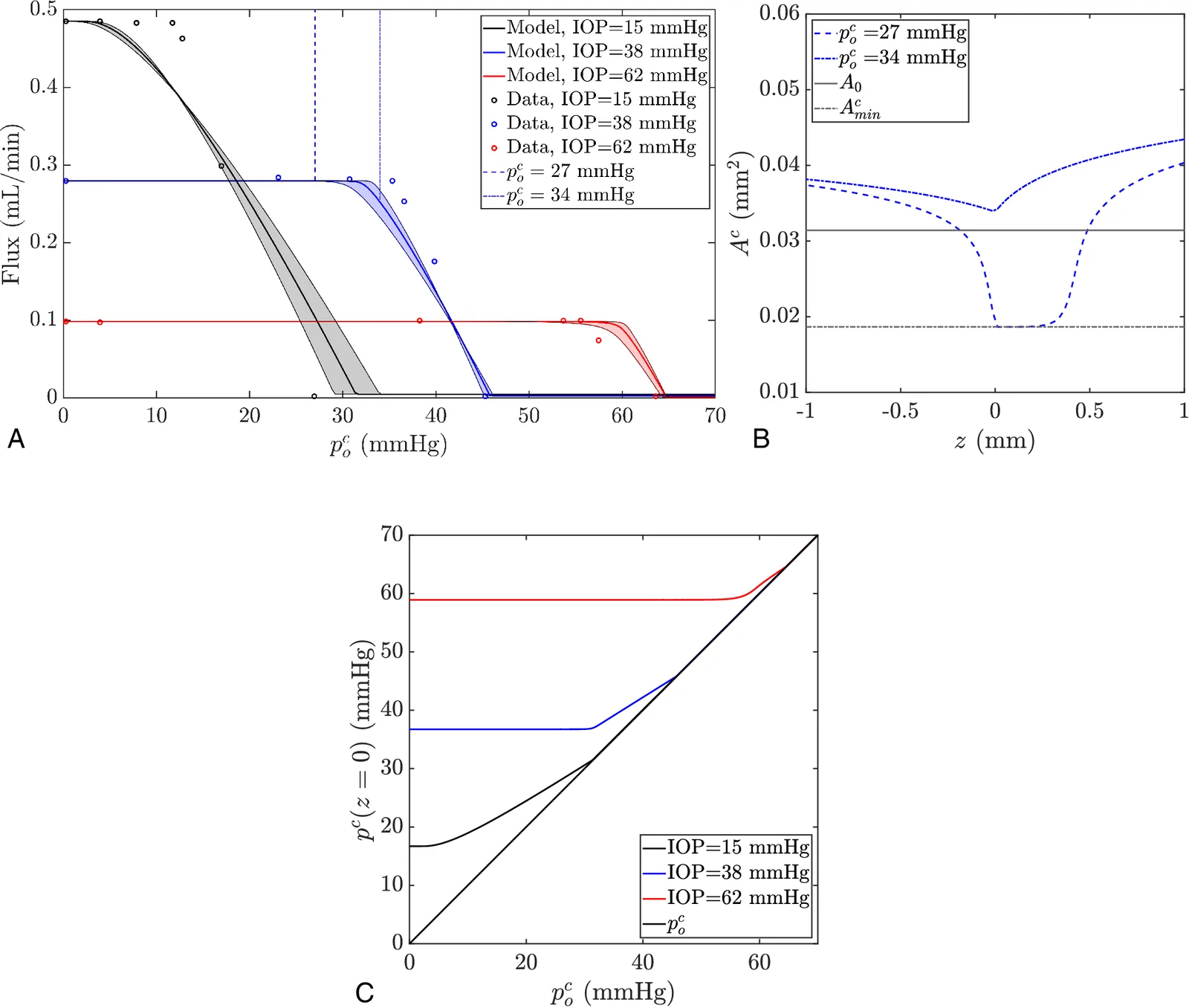
(**A**) Flow rate in the cannulated vortex vein vs. the imposed downstream pressure $p_{o}^{c}$. The *circles* are data points taken from Bill’s Figure 3.^15^ The *solid bold lines* are the results of our model with the parameter values are set as in the Table for rabbits. Changing the reference value of stiffness in the range [0.5*K*_*ref*_, 2*K*_*ref*_] produces curves within the *shaded area*. The extremes of the range are the edges of the *shaded* regions. The vertical lines show the values of $p_{o}^{c}$ corresponding with the area profiles in (**B**). (**B**) Cannulated vessel cross-sectional area profiles *A*^*c*^(*z*) corresponding with the reference model simulation with an IOP of 38 mm Hg (*blue bold line* in (**A**)), for $p_{o}^{c}=27$ mm Hg and $p_{o}^{c}=34$ mm Hg. For $p_{o}^{c}=27$ mm Hg, the vessel is partially collapsed, while for $p_{o}^{c}=34$ mm Hg it is expanded. For a better visualization, we report only the portion of the domain within 1 mm from the boundary between the choroid and the sclera. (**C**) Blood pressure in the cannulated vein at the boundary between choroid and sclera as $p_{o}^{c}$ is artificially raised, for different values of IOP, corresponding with the profiles in (**B**).

To better understand the transition observed when the outlet pressure becomes comparable to IOP, in Figure 5B we plot the cannulated vessel area profiles *A*^*c*^(*z*) for two values of $p_{o}^{c}$ near the transition point, which is close to the IOP. We find that the intrasceleral portion of the vessel does indeed transition from being partially collapsed to its minimal area $A_{min}^{c}$ for outlet pressures below a threshold value close to the IOP (dashed line) to being fully open for greater outlet pressures (dash–dotted line).

Our model simulations also predict the pressure in the cannulated vein at the interface between choroid and sclera during the experiment preformed by Bill. This is shown in Figure 5C, where *p*^*c*^(*z* = 0) attains a constant value, slightly lower than the IOP, when the flux is constant and the vessel is collapsed. As the vessel reopens, the pressure starts to increase, until it reaches the value of the downstream pressure and the flow ceases. Hence, vortex vein collapse is again playing a key role in passively controling the choroidal blood flow.

## Discussion

We have developed a simple theoretical model for blood flow through a vortex vein that successfully captures some of the essential features of the choroidal venous drainage system. The model treats the vortex vein as a compliant vessel, with a segment within the choroid, arranged in series with another segment across the sclera. Much is unknown about vessel collapse here, but in the model we assume that the vein can collapse both in the choroid and in the sclera, according to a tube law relating its cross-sectional area to the transmural pressure difference. The vortex vein is acted on by an external pressure, which is assumed to be constant and equal to the IOP within the choroid, and to decrease linearly across the sclera, from the IOP to the orbital tissue pressure.

The model predicts that the choroidal venous pressure is tightly correlated with the IOP (Fig. 4), reproducing the experimental observation of the constant offset observed between these two pressures as the IOP is increased.^13^^,^^14^ The model predicts that this constant offset results when the pressure gradient along the intrascleral segment of the vein exactly mirrors the external pressure gradient across the sclera; in this configuration, the vortex vein attains a spatially uniform constriction quantified by Equation (4). Indeed, the model elucidates how the tethering of these two pressures is linked to a partial collapse of the vortex vein across the intrascleral segment. For a given IOP, the vessel becomes increasingly collapsed as the flow rate decreases (Fig. 2B), eventually attaining a (spatially uniform) minimal cross-sectional area where the pressure gradient in the blood vessel exactly mirrors that in the external tissue pressure (Fig. 2A). A model reduction suggests that this minimal area, given by Equation (4), is weakly dependent on the flow rate and the length of the intrascleral segment. This minimal value of the vessel cross-sectional area can be translated into a prediction of the small offset between the IOP and the choroidal venous pressure as the vortex vein crosses the sclera. This offset shows that in this configuration the choroidal venous pressure at the inlet to the sclera is slightly lower than the IOP by an amount dependent on the flow rate along the vessel. The pressure in the vortex vein gradually increases as we move distally into the choroidal veins and becomes higher than the IOP at a short distance from the sclera, in agreement with experimental observations.^13^^,^^14^ The predicted close relationship between the the IOP and the choroidal venous pressure is consistent with the usual definition of the choroidal perfusion pressure as the difference between the mean arterial pressure in the ophthalmic artery and the IOP.^44^

Under the assumption that the vortex vein can collapse in the sclera, the model also reproduces the results of rabbit experiments by Bill,^15^ suggesting that a cannulated vortex vein maintains a constant flow rate as the outlet venous pressure is gradually increased to a critical value, approximately equal to the IOP (Fig. 5A); beyond this critical value, the flow rate decreases rapidly to zero. Again, according to our model, the collapse of the vortex vein across the intrascleral segment is the switch that controls this behavior—as the outlet pressure increases, the flow rate is maintained while the vessel is collapsed to its minimal cross-sectional area, but then it immediately begins to decrease once the vortex vein becomes expanded across the sclera (Fig. 5B). These predictions of constant blood flow despite changes in the venous downstream pressure agree with the hypothesis that a hydraulic Starling resistor control mechanism might be in place to control the choroidal venous pressure, first put forward by Bill^15^ and more recently discussed by Spaide et al.^19^ Despite the uncertainty on the value of some of the model parameters, the agreement with Bill’s^15^ results is quite robust with respect to the value of the vessel wall stiffness *K*, the pressure in the orbit *p*_*orbit*_, and the outlet venous pressure.

This concept that choroidal veins are normally in a state of partial collapse implies that they have the capacity to expand. This observation may explain the dilated Haller vessels seen in the pachychoroid syndromes.^45^

In their review, Spaide et al.^19^ discuss a possible alternative mechanism for the correlation between the IOP and the choroidal venous pressure^13^^,^^14^ where vortex vein collapse upon an increase in IOP could be induced by mechanical squeezing from the scleral tissue. The authors notice that the course of the vortex vein through the sclera occurs at an angle similar to that used to make self-sealing incisions during vitrectomy surgery. In these self-sealing incisions, the surgical tunnel closes naturally as the IOP pushes the inner leaf of the incision toward the outer wall of the eye. Spaide et al.^19^ speculated that a similar mechanism may occur with the vortex vein, proposing that an increase in the IOP would increase the force exerted on the inner side of the sclera, thereby squeezing the vein (see Figure 4 in the authors’ paper and related caption). In the Supplementary Material, we present a mechanical argument showing that even a very large increase in the IOP would produce negligible variation in the diameter of the vortex vein. This result suggests that vortex vein collapse is unlikely to be produced by mechanical squeezing from the sclera alone.

Although our model provides useful insight into the mechanics of venous drainage from the choroid, the approach makes a number of simplifying assumptions, such as an elliptical shape of the vessel cross-sectional area during collapse and the collapsibility of both the choroidal and scleral segments of the vessel. Under these hypotheses, when the vessel collapses to its minimal area in the scleral segment, our model well reproduces the experimental observation of the choroidal venous pressure being closely related to the IOP.

In the absence of experiments on humans, we compared our model with Bill’s^15^ experiments on rabbits. The main features of our model are present both in human and rabbit: the presence of the vortex veins subjected to the IOP in the choroid, crossing the sclera at an angle, and subjected to the orbital tissue pressure as they exit the eye. Nevertheless, we are aware of the quantitative differences between humans and rabbits, which are accounted for when reproducing Bill’s setup. The development of a less invasive experimental protocol applicable to humans to investigate the variation of the flux in one vortex in response to increasing downstream pressure would provide data for the validation of our model in humans, but this is challenging. Advances in ultrasound biomicroscopy might provide a direct visualization of the collapse of the vortex veins through the sclera. This would be particularly important to confirm the Starling resistor hypothesis and to investigate the role of vortex vein collapse in the pachychoroid syndromes.^45^

The model offers the possibility for several extensions. In particular, it would be interesting to include an ampulla region, involving the convergence of a large number of chorodial veins at a single point. Furthermore, it would be interesting to explore the interplay with other more active mechanisms of venous flow control, such as those due to the rich innervation of the choroidal blood vessels and the relationship between the IOP and the choroidal blood flow.^46^ Finally, time dependency could be included in the model to explore how oscillatory flows may arise within the vortex veins.^47^

## Supplementary Material

Supplement 1
