# Supplementary material for: Choroidal Venous Pressure Is Passively Controlled by Vortex Vein Collapse: A Theoretical Model
Source: Invest Ophthalmol Vis Sci. 2026 Oct 22;67(12):47. doi: 10.1167/iovs.67.12.47 (PMC13715903; doi:10.1167/iovs.67.12.47)
Supplement: Supplementary file 1 [file iovs-67-12-47_s001.pdf]

Supplementary material

Choroidal venous pressure is passively controlled by vortex veins collapse: a theoretical model

Federica Vanone,[1] Peter S. Stewart,[2†] Alexander J.E. Foss,[3] Tamsin A. Spelman,[4] Jennifer H. Tweedy,[5] Rodolfo Repetto[6]

[1]Gran Sasso Science Institute, L'Aquila, Italy

[2]School of Mathematics & Statistics, University of Glasgow, Glasgow, UK

[3]Department of Ophthalmology, Nottingham University Hospitals NHS Trust, Nottingham, UK

[4]Sainsbury Laboratory, University of Cambridge, Cambridge, UK

[5]Department of Mathematical Sciences, University of Bath, Bath, UK

[6]Department of Civil, Chemical and Environmental Engineering, University of Genoa, Genoa, Italy

[†]Corresponding Author, Peter.Stewart@glasgow.ac.uk

1 Model 1: Derivation of the 'purely viscous' equation of motion

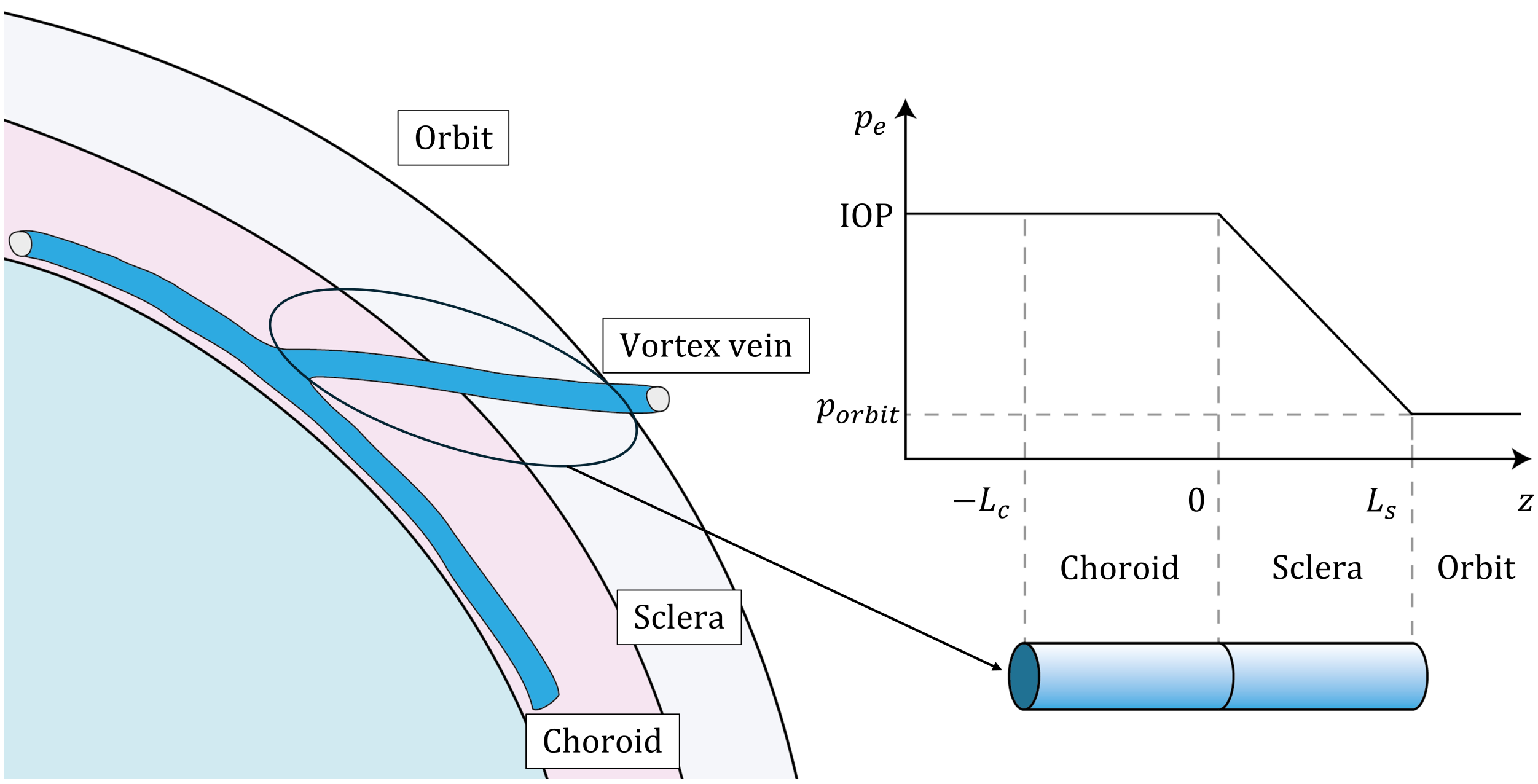

Figure 1: On the left, sketch of the essential elements of the system we are considering: the vortex vein collects blood from the choroidal circulation and drains it out of the eye (orbit). The two parts of the vortex vein we consider in the model are the choroidal and scleral segments. On the right, the domain of the model: the axis of the tube representing the vortex vein starts in $-L_c$ and ends in L_s. The external pressure $p_e(z)$ acting on the tube is IOP in the choroid, and it decreases linearly to p_{orbit} in the sclera.

In the present document, references from the main document are denoted with 'M' preceding the number.

With reference to Figure 1, we describe the vessel using cylindrical polar coordinates (z, r, ϕ) with respect to the axis along the centerline of the vein, and with $z \in [-L_c, L_s]$ being the axial coordinate ($[-L_c, 0]$ inside the choroid, $[0, L_s]$ inside the sclera), where L_c is the length of the choroidal venous segment and L_s is that of the scleral venous segment. The vein is modelled as an axisymmetric tube of length $L_s + L_c$ and variable diameter $d(z)$, so that the radial coordinate $r \in [0, d(z)/2]$ and the angular coordinate $\phi \in [0, 2\pi)$.

1.1 Interstitial flow

Efflux across the sclera is governed by Darcy's law,

$$\mathbf{u}_e = -\frac{k_s}{\mu_w} \nabla p_e, \tag{1}$$

where $\mathbf{u}_e$ (m/s) is the Darcy velocity of the fluid (the flux per unit area of the interstitial fluid outside the vortex vein), k_s (m^2) is scleral permeability and μ_w (Pa·s) is shear viscosity, taken as that of water (the subscript 'e' is used to denote the region external to the vortex vein).

Given that the radius of the eye greatly exceeds the thickness of the scleral tissue, it emerges that the interstitial fluid pressure is approximately linear in the radial coordinate, varying from the intraocular pressure IOP at the boundary with the choroid to the orbital pressure outside the eye. We express this in the form

$$p_e(z) = p_{orbit} + (p_{orbit} - \mathrm{IOP})\frac{(z - L_s)}{L_s}, \quad (0 \le z \le L_s),$$

as plotted in Figure 1.

1.2 Venous flow

We describe the steady flow along the vessel with constant flow rate q, with boundary condition on the pressure at the outlet of the sclera $p|_{z=L_s} = p_o$. The diameter of the vessel d is assumed to be a function of transmural pressure $p - p_e$, with $p(z)$ the pressure in the vessel and $p_e(z)$ (variable) the imposed external pressure

$$p_e(z) = \begin{cases} \mathrm{IOP} & \text{(in the choroid, } -L_c \le z < 0) \\ \mathrm{IOP} - \dfrac{z}{L_s}(\mathrm{IOP} - p_{\mathrm{orbit}}) & \text{(in the sclera, } 0 \le z \le L_s), \end{cases} \tag{2}$$

according to the tube law (M3). The baseline value for the diameter is d_0, which is uniform along the vessel.

The following derivation is performed assuming the tube is expanded with respect to its equilibrium configuration ($A \ge A_0$), thus the cross-section of the tube is assumed circular. We assume axial symmetry of the flow, so that the ϕ-component of the velocity is zero and

$\partial/\partial\phi = 0$ for all variables. The fluid velocity can be written as $\mathbf{u} = u\mathbf{e}_z + v\mathbf{e}_r$, where $u(z,r)$ is the longitudinal component and $v(z,r)$ the radial component.

The unsteady Navier–Stokes equation in the z-direction reads

$$\frac{\partial u}{\partial t} + u\frac{\partial u}{\partial z} + v\frac{\partial u}{\partial r} + \frac{1}{\rho}\frac{\partial p}{\partial z} - \nu\left[\frac{\partial^2 u}{\partial z^2} + \frac{1}{r}\frac{\partial}{\partial r}\left(r\frac{\partial u}{\partial r}\right)\right] = 0, \tag{3}$$

where ρ is the density and ν is the kinematic viscosity of the fluid. Since the flow resembles that in long pipe, we assume that the flow is characterised by two different spatial scales: a characteristic length of longitudinal variations $\mathcal{L}$ and one of radial variations $\mathcal{R}$, so that $\mathcal{R}/\mathcal{L} = \epsilon \ll 1$, and we can assume that $\mathcal{R} \approx \frac{1}{2}d_0$, with d_0 being the baseline value of the tube diameter. Using scalings typical of lubrication theory, we define the following dimensionless variables (denoted with superscript stars) to scale (3):

$$z^* = z/\mathcal{L}, \quad r^* = r/\mathcal{R}, \quad t^* = t\omega, \quad u^* = u/\mathcal{U}, \quad v^* = v/(\epsilon\mathcal{U}), \quad p^* = p/(\rho\nu\mathcal{U}\mathcal{L}/\mathcal{R}^2), \tag{4}$$

where ω is a characteristic frequency of the flow, $\mathcal{U} \approx U_0$ is a characteristic longitudinal velocity whose value is defined in Table M1, and we have introduced the dynamic viscosity $\mu = \rho\nu$. The dimensionless form of (3) reads

$$\alpha^2\frac{\partial u^*}{\partial t^*} + \epsilon\mathrm{Re}\left(u^*\frac{\partial u^*}{\partial z^*} + v^*\frac{\partial u^*}{\partial r^*}\right) + \frac{\partial p^*}{\partial z^*} - \epsilon^2\frac{\partial^2 u^*}{\partial z^{*2}} - \frac{1}{r^*}\frac{\partial}{\partial r^*}\left(r^*\frac{\partial u^*}{\partial r^*}\right) = 0, \tag{5}$$

where we have introduced the Reynolds number $\mathrm{Re} = \mathcal{U}\mathcal{R}/\nu$ and the Womersley number $\alpha = \sqrt{\mathcal{R}^2\omega/\nu}$, with ω representing the angular frequency of the flow, which, in this case, is the frequency of the heart beat ($\omega \approx 1$ Hz). In the present application, as discussed in the main document, the Reynolds number is of order 1 to 10. Moreover, assuming $d_0 = 250\ \mu$m and $\nu = 4.2 \cdot 10^{-6}$ m$^2/s$, we obtain $\alpha^2 \approx 0.0037$ (see Table M1). With such a small value of the Womersley number the flow can be thought of as quasi-steady i.e. at every time instance the flow can be regarded as steady and so we neglect time derivatives. This argument is also confirmed by calculation of the time required for a pipe flow to adapt to the instantaneous value of the pressure gradient, which is of order $\rho d_0^2/(4\mu\alpha_1^2) \approx 7 \cdot 10^{-4}$ s,[1] with $\alpha_1 \approx 2.4$ the first zero of the Bessel function J_0. This is indeed much faster than the heart beat. The value of ϵ depends on the way the vessel collapses, but in all cases considered, we have $\epsilon \ll 1$. This allows us to neglect inertia in the Navier–Stokes equation, and we will verify the correctness of this assumption in §3. Using the same reasoning, it is possible to show that the Navier–Stokes equation in the radial direction implies that the pressure does not depend on r.

Returning to dimensional variables, we can thus simplify (3) to obtain

$$\frac{\mathrm{d}}{\mathrm{d}r}\left(r\frac{\mathrm{d}u}{\mathrm{d}r}\right) = \frac{r}{\mu}\frac{\mathrm{d}p}{\mathrm{d}z}. \tag{6}$$

By integrating this equation twice with respect to r, imposing regularity at $r = 0$ and the no-slip boundary condition at $r = d/2$, we obtain the velocity distribution

$$u(z,r) = \frac{1}{4\mu}\left[r^2 - \left(\frac{d}{2}\right)^2\right]\frac{\mathrm{d}p}{\mathrm{d}z}. \tag{7}$$

The corresponding flow rate at every cross-section of the vessel is given by

$$q(z) = \iint_A u(z,r)\,\mathrm{d}A = 2\pi \int_0^{d/2} u(z,r)\,\mathrm{d}r = -\frac{\pi}{8\mu}\frac{\mathrm{d}p}{\mathrm{d}z}\left(\frac{d}{2}\right)^4. \tag{8}$$

The continuity equation in cylindrical coordinates reads

$$\frac{\partial u}{\partial z} + \frac{1}{r}\frac{\partial}{\partial r}(rv) = 0. \tag{9}$$

By integrating over the vessel cross-section and imposing $v(z, r = d/2) = 0$ (the kinematic condition), we find that the flow rate must be constant along the vessel i.e. with respect to z, as

$$\frac{\mathrm{d}q}{\mathrm{d}z} = 0, \tag{10}$$

and so using equation (8) we obtain

$$\frac{\mathrm{d}}{\mathrm{d}z}\left[\frac{\mathrm{d}p}{\mathrm{d}z}\left(\frac{d}{2}\right)^4\right] = 0. \tag{11}$$

Integrating leads to

$$\frac{\mathrm{d}p}{\mathrm{d}z}\left(\frac{d}{2}\right)^4 = -\frac{8\mu}{\pi}q, \tag{12}$$

and writing $A = \pi\left(\frac{d}{2}\right)^2$ we obtain equation (M1)

$$\frac{\mathrm{d}p}{\mathrm{d}z} = -\frac{8\mu\pi}{A^2}q = -\mathcal{R}q, \tag{13}$$

where $\mathcal{R}$ is a the friction term.

The flow rate q is imposed at the tube inlet ($z = -L_c$) and the pressure at the outlet p_o ($z = L_s$).

For the derivation in the collapsed case ($A < A_0$), we refer to Pedley,[2] who dropped the assumption of axial symmetry, assumed that during collapse the tube cross-section is an ellipse, with semi-axes a and b and applied lubrication theory. The equation they obtain has the same structure as (13), $\frac{\mathrm{d}p}{\mathrm{d}z} = -\mathcal{R}q$, with the following expression for friction:

$$\mathcal{R} = \frac{4\mu}{\pi}\frac{a^2 + b^2}{a^3 b^3}. \tag{14}$$

For practical purposes, an approximation of $\mathcal{R}$ as a power laws of the area is commonly used, such as $\mathcal{R} \approx \dfrac{8\mu\pi A_0^{1/2}}{A^{5/2}}$.[3] A different approximation is proposed by Cancelli and Pedley[4] $\mathcal{R} \approx \dfrac{8\mu\pi A_0}{A^3}$, which can be derived using the assumption of small area changes with constant value of $a + b$.[5] Numerical investigations show that Cancelli and Pedley's approximation performs better under the assumption of the elliptical cross-section collapsing with constant perimeter, while Brook *et al.*'s approximation is more suitable when a is constant during collapse. In our work, we choose the approximation by Brook *et al.*[3]

2 Model 2: Fully non-linear 1D model

In this section we introduce a fully non linear model to describe fluid flow in the vortex vein that also accounts for inertial terms that have been neglected to derive equation (13).

We model the vortex vein as a collapsible tube and assume the blood flow within it is one-dimensional and steady. Under these conditions the cross-sectionally averaged continuity equation imposes that the volumetric flow rate q is constant along the tube, and under the assumption of axis-symmetry, the momentum conservation equation can be written as:[6]

$$\frac{4}{3}\frac{\mathrm{d}}{\mathrm{d}z}\left(\frac{q^2}{A}\right) + \frac{A}{\rho}\frac{\mathrm{d}p}{\mathrm{d}z} + \frac{8\pi\mu}{\rho}\frac{q}{A} = 0. \tag{15}$$

In the above equation, $A(z)$ represents the cross-sectional area of the tube, and $p(z)$ the fluid pressure, which is assumed to be constant within a cross-section of the vein. The coefficient 4/3 and the specific expression of the viscous term follow from the assumption that the velocity satisfies Poiseuille's law, which is an appropriate choice in the case of low Reynolds number steady flows, and is consistent with the derivation reported in §1.

Combining equation (15) with the tube law (M3), we obtain a nonlinear first-order differential equation for the variable $A(z)$, which is solved imposing the flow rate q and the downstream pressure $p(z = L_s) = p_o$.

The nonlinear differential equation is discretised using a second-order finite difference scheme and solved using the `fsolve` Matlab[7] function following Stewart *et al.*[8]

3 Comparison between models 1 and 2

To check whether neglecting inertia in the equation for fluid flow (M1) is a reasonable assumption, we compare the results obtained with the purely viscous model (§ 1) and the fully nonlinear one-dimensional model (§ 2). Since the fully nonlinear model is axis-symmetric, in order to facilitate a comparison we assume the same also holds for the purely viscous model and use the friction term corresponding to a circular cross section for all values of A: $\mathcal{R} = \frac{8\mu\pi}{A^2}$. In Figure 2 we show pressure profiles analogous to those reported in Figure M2(a) of the main text, obtained with different values of the flow rate. Dashed lines are produced with the model with no inertia and solid lines with the fully nonlinear model. The figure shows that the agreement of the two models is invariably very good, even for the largest value of the flow rate considered, which corresponds to a Reynolds number significantly higher than in physiological conditions.

Comparison of the inlet choroidal pressure shows that the largest difference between the two models is ≈ 0.05 %. This justifies the use of the simpler model with no inertia to produce all results presented in the main text.

4 Parameter values

As baseline diameter of the human vortex vein, we take the value of $d_0 = 250$ μm reported by Cai et al.[9]

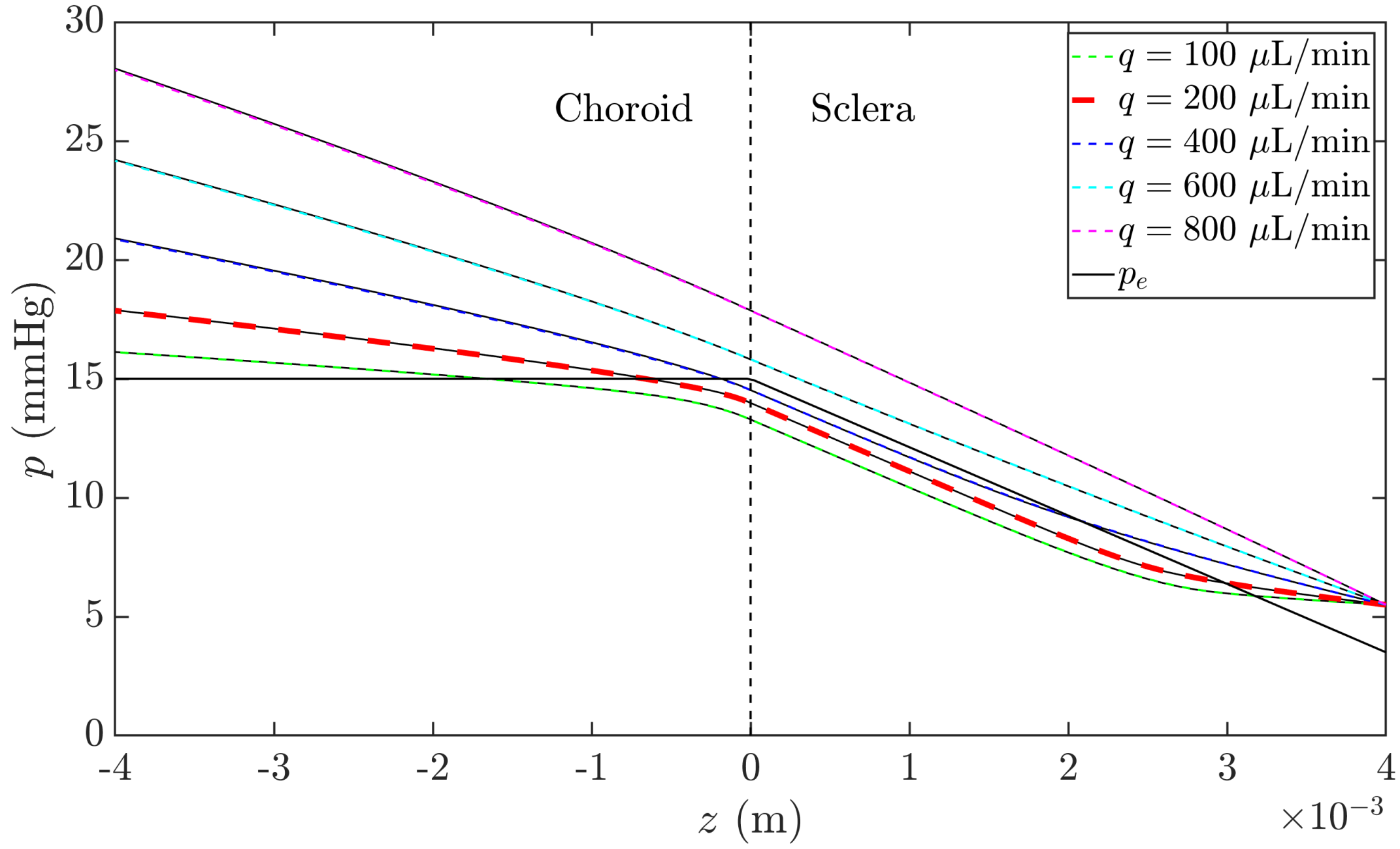

Figure 2: Pressure profiles along the vortex vein, computed for different values of the flow rate q. Dashed lines are produced with the inertialess, axis-symmetric model and solid lines with the fully nonlinear model. The other parameters values are set as in Table M1 for human.

Blood velocity in the vortex vein has been measured with a Doppler technique by Abdallah et al.,[10] who report a velocity of $U_0 = 7$ cm/s, thus the corresponding flow rate can be estimated as $q = U_0 A_0 = \frac{1}{4}\pi d_0^2 U_0 \approx 200\ \mu$L/min, where A_0 is the cross-sectional area of the vessel. If we assume in the eye there are four vortex veins,[11,12] this estimate agrees with the flow rate in one vortex veins being 170–400 μL/min, computed by assuming choroidal blood flow is 20 times retinal blood flow.[13–15] We use this value as baseline for blood flow rate in a single vortex vein.

Bill[16] measured a flow rate in a single vortex vein of 500 μL/min in rabbit eyes, at IOP of 15 mmHg. This value is higher than our estimate for a human, so we use this large value when reproducing Bill's experiments as described in § 'Modifications to the model to reproduce Bill's[16] experiment'.

The thickness of the sclera at the equator of eye is 0.4 mm in humans[17] and 0.2 - 0.25 mm in rabbits.[18] The length of a vortex vein through the human sclera is 4 mm.[19] Therefore, it seems reasonable to assume the length of the vortex vein through the rabbit sclera is 2.5 mm.

The portion of the vein we consider inside the choroid in our simulations, has the same length as the portion in the sclera ($L_c = L_s$); we note that the flow across the scleral segment of the vein is insensitive to the value of L_c.

For the orbit tissue pressure, the review by Enz and Tschopp[20] reports values in the range [2.5, 6] mmHg in healthy humans. Therefore, it seems reasonable to assume $p_{orbit} = 3.5$ mmHg both in human and rabbit, where the measurements are very uncertain.[21]

Tube law parameters Blood flow through collapsible veins has been well studied in other parts of the body,[3,22,23] where the typical approach is to assume an empirical tube law to describe the elastic response of the vessel, writing the transmural (internal-external) pressure as a function of the vessel cross-sectional area (M3).

Such constitutive laws have been constructed based on both experiments[24–27] and theoretical studies of the buckling of an elastic ring;[28] across these approaches it emerges that when the conduit is collapsed the transmural pressure across the wall of the flexible vessel is proportional to $A^{-3/2}$.[25,29,27] In this case the constant of proportionality depends linearly on the Young's modulus of the material and the cube of the ratio of the wall thickness to the lumen radius.[28]

As discussed above, it is common to approximate the vessel wall mechanics by assuming a constitutive law which writes the transmural pressure $p - p_e$ as a function of the vessel cross-sectional area A scaled relative to the cross-section of the undeformed circular vessel A_0. Hence, the transmural pressure can be expressed as[25]

$$p - p_e = K\mathcal{P}\left(\frac{A}{A_0}\right). \tag{16}$$

Although the tube law function $\mathcal{P}$ can be expressed as a piecewise function depending on the state of inflation/collapse of the vessel,[5,2] we instead express the law as a sum of two power law functions across the entire range of scaled cross-sectional areas $\alpha = A/A_0$, in the form

$$\mathcal{P}(\alpha) = \alpha^m - \alpha^{-n}, \tag{17}$$

where the first term dominates the response during inflation, and the second one during collapse.[29,30] In line with our discussion about vessel collapse, we choose $n = 3/2$[28,25,29] and consider $m = 10$ consistent with previous studies in veins.[3,22,23]

It remains to evaluate the constant of proportionality, K. In their work using similar tube laws in lung physiology Elad and coworkers[29,30] estimate this stiffness parameter directly using experiments. Alternatively, it is also common to estimate the constant of proportionality using the critical buckling pressure of a linearly elastic beam, postulated by McClurken *et al.*[31] and used in subsequent studies.[3,22,23,6] In this case we set

$$K = \frac{E}{12(1-\nu^2)}\left(\frac{h}{r_0}\right)^3, \tag{18}$$

where E is the Young's modulus of the tissue, ν is the Poisson's ratio of the material, $r_0 = d_0/2$ is the radius of the undeformed vessel and h/r_0 is the wall thickness to lumen radius ratio when the tube is its baseline (circular) configuration. The remaining challenge is to estimate or measure the individual parameters which form this stiffness constant. Since biological tissue is almost perfectly incompressible, we set $\nu = 0.5$ throughout.[31] To the best of our knowledge, there are no direct measurements of the Young's modulus or the wall thickness to lumen radius ratio for human vortex veins. However, the human central retinal vein (CRV) is of comparable size and composition to the vortex veins, where the Young's modulus is estimated $E = 0.6$ MPa[32] and the ratio of the wall thickness to lumen radius is approximately 10%;[6] the latter is comparable to values of this ratio reported elsewhere in the body.[33] These values translates into a stiffness parameter $K_{ref} = 66$ Pa.

In this study we consider a baseline value of $K = K_{ref}$ based on the stiffness estimate for the CRV, but, since there is uncertainty in the value, we consider a range between $K = 13$ Pa and $K = 330$ Pa.

5 Iterative procedure for solving the problem in 4 vortex veins

In § Modifications to the model to reproduce Bill's[16] experiment we extend our model to reproduce experiments where the downstream pressure is increased in the cannulated vortex vein (1 out of 4) and the flux is measured. The downstream pressure is known in all the veins: it takes the value in Table 1 in the undisturbed veins, and a prescribed value in the cannulated vein. The sum of the fluxes in the 4 veins is assumed to have a known value, Q, while the flux in the cannulated vein, Q^c, is unknown. Since the vortex veins are connected by low resistance shunts at the inlet of the choroid, we impose the constraint of all the veins having the same inlet pressure $p(z = -L_s)$.

We define a function $[p^c, p^u] = g(Q^c, p_o^c, p_o^u)$ that takes in input the flux in the cannulated vein, Q^c, the outlet pressure in the cannulated vein p_o^c, and the outlet pressure in each of the undisturbed veins, p_o^u. The function g computes the pressure profile in the cannulated vein by solving equation (13) with flux $q(z = -L_s) = Q^c$ and $p(z = L_s) = p_o^c$ as boundary condition on the pressure using Matlab ODE solver `ode15s`. Then, it computes the pressure profile in each of the undisturbed veins with $q(z = -L_s) = \frac{Q-Q^c}{3}$ as boundary condition on the flux and $p(z = L_s) = p_o^u = p_o$ as condition on the pressure. The output of the function is a vector containing the inlet pressure in the cannulated vein, p^c, and in the undisturbed veins, p^u.

This function is used in the following iterative procedure, whose target is to find Q^c such that $p^c = p^u$, which is the constraint we impose on the inlet pressure:

tol $\leftarrow 10^{-5}$
Define $[Q_a^c, Q_b^c]$ such that $Q_a^c < Q_b^c < Q$ and $\left|\frac{Q_a^c - Q_b^c}{Q_a^c}\right| > \text{tol}$
Compute $[p_a^c, p_a^u] = g(Q_a^c, p_o^c, p_o^u)$, $[p_b^c, p_b^u] = g(Q_b^c, p_o^c, p_o^u)$
Require $(p_a^c - p_a^u)(p_b^c - p_b^u) < 0$. If not, try another $[Q_a^c, Q_b^c]$
while $\left|\frac{Q_a^c - Q_b^c}{Q_a^c}\right| > \text{tol}$ **do**
  $Q_{mean}^c = \frac{Q_a^c + Q_b^c}{2}$
  $[p_{mean}^c, p_{mean}^u] = g(Q_{mean}^c, p_o^c, p_o^u)$
  if $(p_{mean}^c - p_{mean}^u)(p_a^c - p_a^u) > 0$ **then**
    $Q_a^c = Q_{mean}^c$
  else
    $Q_b^c = Q_{mean}^c$
  end if
end while

6 Robustness of the results with respect to the parameters

We test the robustness of the reproduction of Bill's results[16] by our model to changes in the parameters.

Figure M5a in the main text shows the sensitivity to the vessel stiffness K. This parameter affects the behaviour of the model, but for values of K in the range $[0.5K_{ref}, 2K_{ref}]$, the flux in the cannulated vein in response to changes in its downstream pressure is captured by our model.

The model is even less sensitive to the pressure in the orbit, which we test in the range $[1.75, 7]$ mmHg, thus changing it by a factor of two in both directions (Figure 3a). Increasing the orbit tissue pressure results in a shift of the curve to the left: the flux starts decreasing earlier than in the reference case, but with the same slope. The effect is only noticeable for IOP= 15 mmHg. A little effect, only detectable for IOP= 15 mmHg is produced also by the change in the downstream pressure in the undisturbed veins p_o^u, which we vary in the range $[p_{orbit}, p_{orbit} + 4\text{mmHg}]$ (Figure 3b). In this case, increasing p_o^u results in a slightly steeper curve.

We have checked that the plots for the reference set of parameters (bold lines in Figure M5a) are indistinguishable if we change the friction term to the axis-symmetric friction $\mathcal{R} = \frac{8\mu\pi}{A^2}$ for all A.

7 Compression of the vortex vein upon increase in the IOP

In this section, we quantify the compression of the vortex vein due to squeezing of the scleral tissue upon increase in the IOP. This has been proposed in a review by Spaide *et al.*[34] as an alternative mechanism for the correlation between choroidal venous pressure and IOP.

The IOP is related to the globe volume V through ocular compliance, $C = \frac{\mathrm{d}V}{\mathrm{d(IOP)}}$, which is typically assumed to be constant and equal to 1 μL/mmHg.[35] Thus, assuming that the scleral tissue is incompressible, it is possible to compute the change in scleral thickness Δt corresponding to an increase in IOP, Δ(IOP). The variation of scleral thickness can be easily related to the decrease in vortex vein diameter, if the angle at which the vessel exits the eye is known. Assuming that the eye is spherical and that the sclera is thin compared to the radius of the eye bulb one obtains that

$$\frac{\Delta t}{\Delta(\mathrm{IOP})} \approx \frac{Ct}{2\pi R^3} \approx 0.05\mu\mathrm{m}\,\mathrm{mmHg}^{-1}, \tag{19}$$

where t and R are the reference values of scleral thickness and radius of the eye bulb. This is a tiny variation of the thickness of the sclera, even for a very large increase in IOP, which would produce negligible variation in the diameter of the vortex vein. This simple argument suggests that vortex vein collapse alone is unlikely to be produced by mechanical squeezing from the sclera.

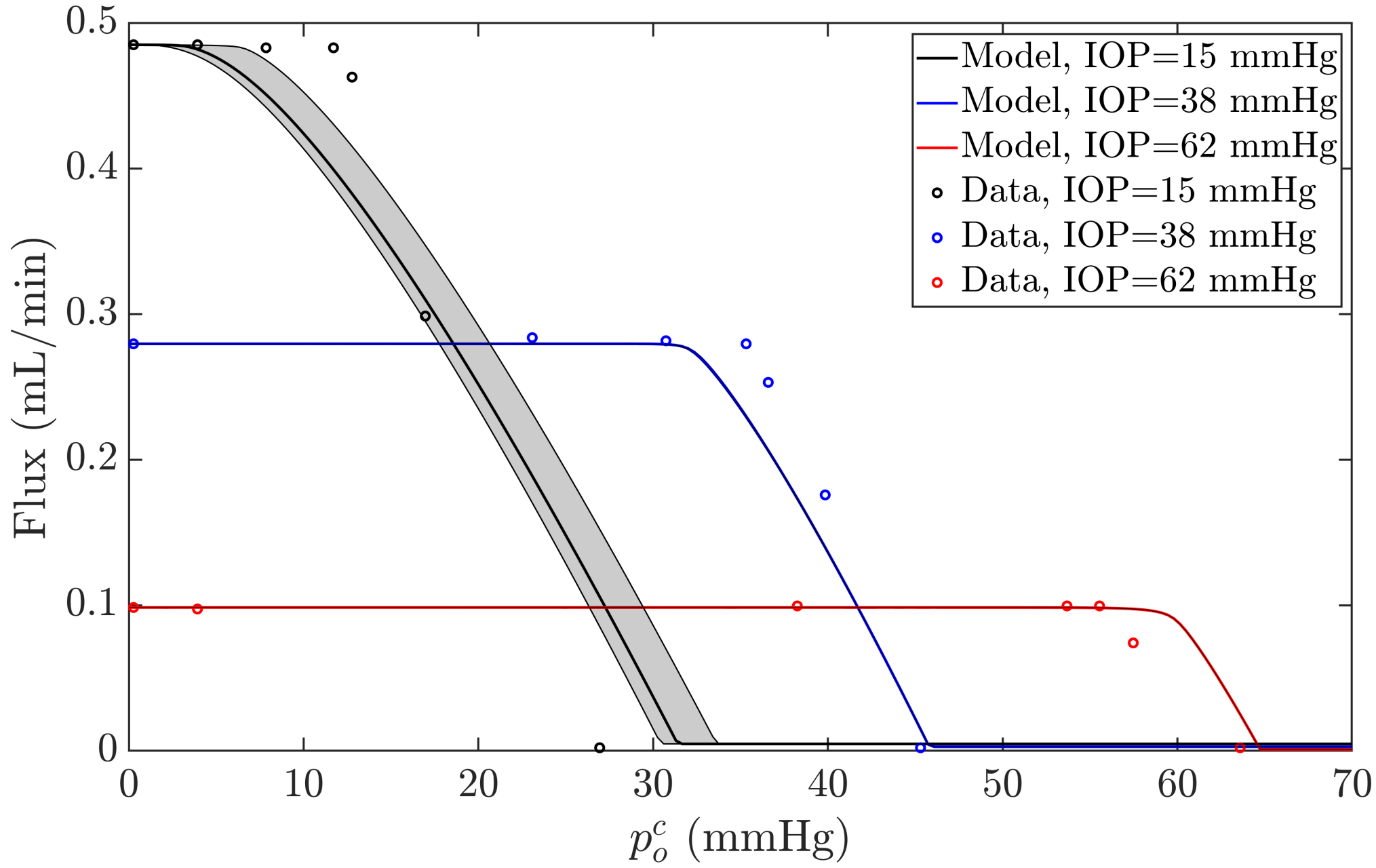

(a)

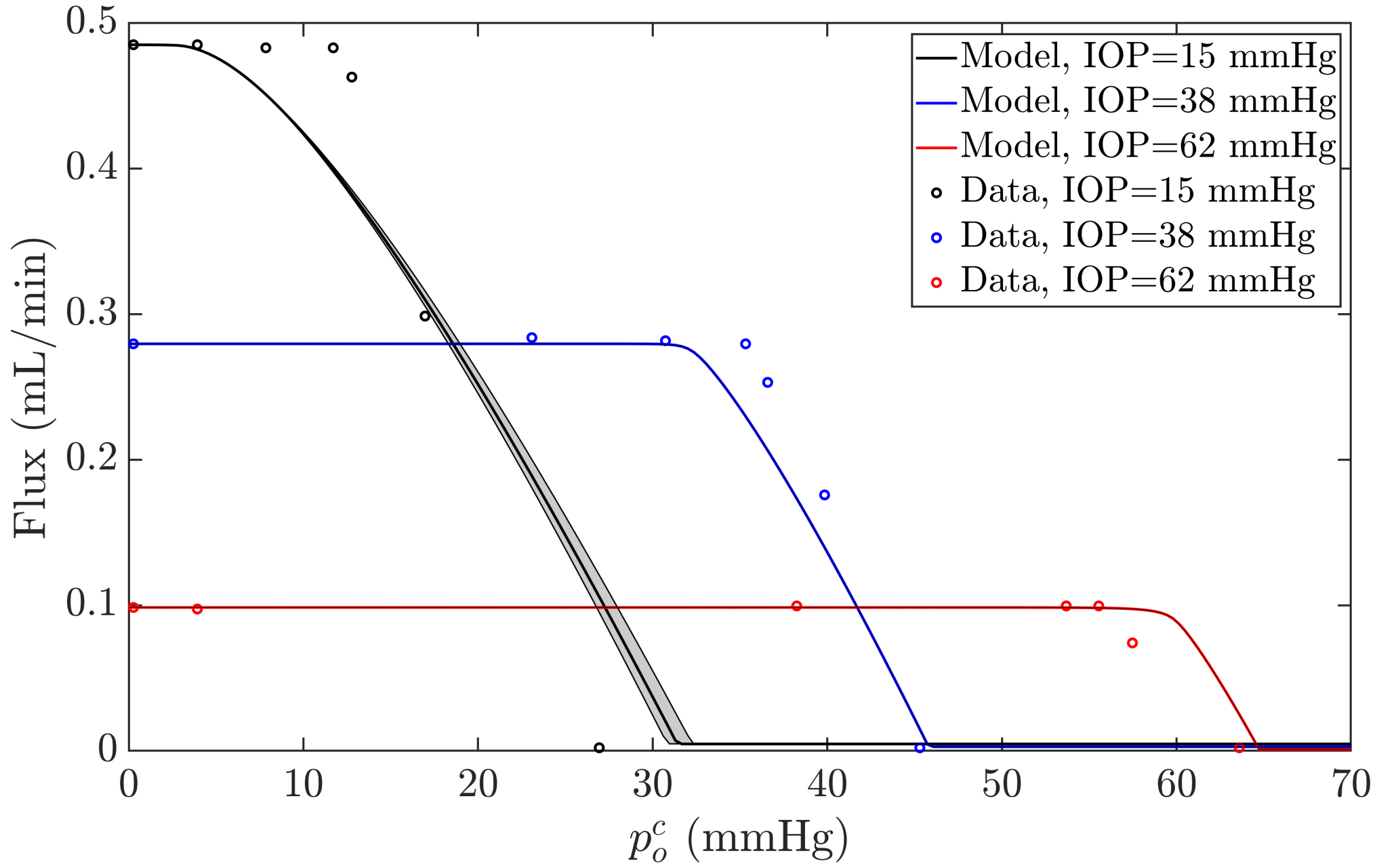

(b)

Figure 3: Blood flow in the cannulated vortex vein as its downstream pressure p_o^c is artificially increased. Solid lines are the results of our model, compared with Bill's[16] data (circles). The bold lines are obtained with the reference set of parameters for rabbits (Table 1). The shaded area in (a) is obtained varying by a factor of 2 the orbit tissue pressure p_{orbit}, thus in the range $[1.75, 7]$. The shaded area in (b), is obtained by considering p_o^u in the range $[p_{orbit}, p_{orbit} + 4\text{mmHg}]$. The extremes of the considered ranges are the edges of the shaded regions.

References

[1] Pozrikidis C. *Fluid dynamics: theory, computation, and numerical simulation*. Springer Science & Business Media, 2009.

[2] Pedley TJ. *The fluid mechanics of large blood vessels*. University Press, 1980.

[3] Brook BS, Falle SAEG, and Pedley TJ. Numerical solutions for unsteady gravity-driven flows in collapsible tubes: evolution and roll-wave instability of a steady state. *J Fluid Mech*, 396:223–256, 1999.

[4] Cancelli C and Pedley TJ. A separated-flow model for collapsible-tube oscillations. *Journal of Fluid Mechanics*, 157:375–404, 1985.

[5] Wild R, Pedley TJ, and Riley DS. Viscous flow in collapsible tubes of slowly varying elliptical cross-section. *J Fluid Mech*, 81(2):273–294, 1977.

[6] Spelman TA and Stewart PS. Shock wave propagation along the central retinal blood vessels. *Proc A*, 476(2234):20190269, 2020.

[7] The MathWorks Inc. Matlab version: 24.1 (r2024a), 2024.

[8] Stewart PS, Waters SL, and Jensen OE. Local and global instabilities of flow in a flexible-walled channel. *European Journal of Mechanics-B/Fluids*, 28(4):541–557, 2009.

[9] Cai CX, Xiong XM, Li T, et al. Vortex vein engorgement and different shapes of venous drainage systems in polypoid choroidal vasculopathy vs. age-related macular degeneration on indocyanine green angiography. *Exp Ther Med*, 25(4):162, 2023.

[10] Abdallah W, Fawzi A, Patel H, et al. Blood velocity measurement in the posterior segment of the rabbit eye using combined spectral doppler and power doppler ultrasound. *Graefes Arch Clin Exp Ophthalmol*, 248:93–101, 2010.

[11] Lim MC, Bateman JB, and Glasgow BJ. Vortex vein exit sites: Scleral coordinates. *Ophthalmology*, 102:942–946, 1995.

[12] Kutoglu T, Yalcin B, Kocabiyik N, and Ozan H. Vortex veins: anatomic investigations on human eyes. *Clin Anat*, 18:269–273, 2005.

[13] Sayah DN, Descovich D, Costantino S, and Lesk MR. The association between the pulsatile choroidal volume change and ocular rigidity. *Ophthalmol Sci*, 4(6):100576, 2024.

[14] Riva CE, Grunwald JE, Sinclair SH, and Perrig BL. Blood velocity and volumetric flow rate in human retinal vessels. *Invest Ophthalmol Vis Sci*, 26:1124–1132, 1985.

[15] Feke GT, Tagawa H, Deupree DM, Goger DG, Sebog J, and Weirer JJ. Blood flow in the normal human retina. *Invest Ophthalmol Vis Sci*, 30:58–65, 1989.

[16] Bill A. Aspects of the regulation of the uveal venous pressure in rabbits. *Exp Eye Res*, 1(3):193–199, 1962.

[17] Olsen TW, Aaberg SY, Geroski DH, and Edelhauser HF. Human sclera: Thickness and surface area. *Am J Ophthalmol*, 125:237–241, 1998.

[18] Peiffer JRL, Pohm-Thorsen L, and Corcoran K. Chapter 19 - models in ophthalmology and vision research. In Manning PJ, Ringler DH, and Newcomer CE, editors, *The Biology of the Laboratory Rabbit*, American College of Laboratory Animal Medicine, pages 409–433. Academic Press, San Diego, 2 edition, 1994.

[19] Imanaga N, Terao N, Nakamine S, et al. Scleral thickness in central serous chorioretinopathy. *Ophthal Retina*, 5:285–291, 2021.

[20] Enz TJ and Tschopp M. Assessment of orbital compartment pressure: A comprehensive review. *Diagnostics*, 12:1481, 2022.

[21] Forrest CR, Khairallah E, and Kuzon WMJ. Intraocular and intraorbital compartment pressure changes following orbital bone grafting: a clinical and laboratory study. *Plast Reconstr Surg*, 104(1):48–54, 1999.

[22] Müller LO and Toro EF. A global multiscale mathematical model for the human circulation with emphasis on the venous system. *Int J Numer Method Biomed Eng*, 30(7): 681–725, 2014.

[23] Contarino C, Toro EF, Montecinos GI, Borsche R, and Kall J. Junction-generalized riemann problem for stiff hyperbolic balance laws in networks: an implicit solver and ader schemes. *J Comp Phys*, 315:409–433, 2016.

[24] Moreno AH, Katz AI, Gold LD, and Reddy RV. Mechanics of distension of dog veins and other very thin-walled tubular structures. *Circ Res*, 27(6):1069–1080, 1970.

[25] Shapiro AH. Steady flow in collapsible tubes. *J Biomech Eng*, 99(3):126–147, 1977.

[26] Kececioglu I, McClurken ME, Kamm RD, and Shapiro AH. Steady, supercritical flow in collapsible tubes. part 1. experimental observations. *J Fluid Mech*, 109:367–389, 1981.

[27] Fung YC. *Biomechanics: Circulation*. Springer Science & Business Media, 1997.

[28] Flaherty JE, Keller JB, and Rubinow SI. Post buckling behavior of elastic tubes and rings with opposite sides in contact. *SIAM J Appl Math*, 23(4):446–455, 1972.

[29] Elad D, Kamm RD, and Shapiro AH. Choking phenomena in a lung-like model. *J Biomech Eng*, 109(1):1–9, 1987.

[30] Elad D, Kamm RD, and Shapiro AH. Mathematical simulation of forced expiration. *J Appl Physiol*, 65(1):14–25, 1988.

[31] McClurken ME, Kececioglu I, Kamm RD, and Shapiro AH. Steady, supercritical flow in collapsible tubes. part 2. theoretical studies. *J Fluid Mech*, 109:391–415, 1981.

[32] Guidoboni G, Harris A, Cassani S, et al. Intraocular pressure, blood pressure, and retinal blood flow autoregulation: a mathematical model to clarify their relationship and clinical relevance. *Invest Ophthalmol Vis Sci*, 55(7):4105–4118, 2014.

[33] Caro CG, Pedley TJ, Schorter RC, and Seed WA. *The mechanics of the circulation.* Cambridge University Press, 2012.

[34] Spaide RF, Cheung CMG, Matsumoto H, et al. Venous overload choroidopathy: a hypothetical framework for central serous chorioretinopathy and allied disorders. *Prog Retin Eye Res*, 86:100973, 2022.

[35] Ethier CR, Johnson M, and Ruberti J. Ocular biomechanics and biotransport. *Annu Rev Biomed Eng*, 6(1):249–273, 2004.
